# Evaluation of Diagnostic Performance of Three Commercial Interferon-Gamma Release Assays for *Mycobacterium tuberculosis*

**DOI:** 10.3390/diagnostics14192130

**Published:** 2024-09-25

**Authors:** Richard Kutame, Gifty Boateng, Yaw Adusi-Poku, Felix Sorvor, Lorreta Antwi, Florence Agyemang-Bioh, Bright Ayensu, Vincent Gyau-Boateng, Franklin Asiedu-Bekoe

**Affiliations:** 1 National Public Health and Reference Laboratory, Harley Street, Korle Bu Teaching Hospital, Accra P.O. Box GP300, Ghana; gifty.boateng@ghs.gov.gh (G.B.); lorreta.antwi@ghs.gov.gh (L.A.); florence.agyemang-bioh@ghs.gov.gh (F.A.-B.); bright.ayensu@ghs.gov.gh (B.A.); vg.boateng77@gmail.com (V.G.-B.); 2 National TB Control Programme, Korle Bu Teaching Hospital, Accra P.O. Box KB493, Ghana; adusipokuyaw@gmail.com (Y.A.-P.); felix.sorvor@ghs.gov.gh (F.S.); 3 Public Health Division, Ghana Health Service, Korle Bu Teaching Hospital, P.M.B. Ministries, Accra P.O. Box MB582, Ghana; franklin.asiedubekoe@ghs.gov.gh

**Keywords:** IGRA, interferon gamma, evaluation, tuberculosis, diagnosis, *Mycobacterium tuberculosis*, SD biosensor

## Abstract

Interferon-gamma release assays (IGRAs) have gained attention for the diagnosis of latent tuberculosis infection (LTBI) due to their higher specificity compared to the tuberculin skin test (TST). However, the IGRA’s performance varies across different populations. This study evaluated the diagnostic performance of three IGRAs (TBF-FIA, TBF-ELISA, and QFT-Plus) in Ghana, comparing them among individuals exposed and unexposed to MTB infection. Conducted in TB clinics across three regions, this prospective and cross-sectional study included healthy individuals with no known TB exposure (unexposed group) and patients with confirmed active TB (exposed group). Blood samples were tested using all three assays as per the manufacturers’ guidelines. The TBF-ELISA showed 3.4% higher sensitivity but 4.6% lower specificity compared to QFT-Plus. The TBF-FIA had sensitivity of 78.5–87.3% and specificity of 82.9–90.0%. These findings indicate that while the three IGRAs offer similar diagnostic accuracy, the variations in specificity and limited data on assays like TBF-FIA require further investigation.

## 1. Introduction

Despite the significant mortality amounting to over one million deaths annually [1], tuberculosis (TB) caused by *Mycobacterium tuberculosis* (MTB) can remain hidden within individuals with latent TB infection (LTBI). Identifying these individuals and prioritizing preventive treatment for those at high risk of developing active TB is essential for TB elimination [2]. Based on the WHO policy, two immunological tests can be used to diagnose LTBI, namely the tuberculin skin test (TST) and interferon-gamma release assays (IGRAs) [3]. However, both tests indirectly assess a past or ongoing immune response to MTB antigens and do not directly confirm the presence of live bacteria [4].

The tuberculin skin test (TST) is an in vivo diagnostic tool that relies on the intracutaneous injection of a mixture containing a standardized purified protein derivative (PPD-S) from MTB, as well as a PPD from atypical Mycobacterium. A delayed-type hypersensitivity reaction at the injection site indicates prior exposure to these antigens [5]. The TST’s complex antigen mixture lacks specificity for MTB, leading to false-positive reactions from prior BCG vaccination, exposure to NTMs, or past *M. tuberculosis* infections [6].

IGRAs, in contrast to the TST, utilize MTB-specific antigens (ESAT-6, CFP-10, and TB7.7) obtained from region of difference 1 (RD1) of the MTB genome, which are absent in BCG and most NTMs, with some exceptions, and this enhances their specificity for *M. tuberculosis* infection [7,8].

A review of the extant literature suggests a growing body of knowledge on the performance of IGRAs and their utility in the management of LTBI, and we present a few pertinent findings. Some studies have suggested that IGRAs offer comparable sensitivity to TST in diagnosing latent tuberculosis infection (LTBI), while demonstrating greater specificity [9]. Additionally, IGRA results appear to correlate more directly with the degree of *M. tuberculosis* exposure [10]. An IGRA was also reported to have a high positive detection rate (86.7%) compared with other methods, including the TST (66.7%), among children with TB, and a significantly lower positive rate (6.7%) than the TST (76.7%) among vaccinated children. Additionally, the specificity of IGRAs was found to be better (86.7%) than that of the TST (40%) [11]. In another study among various risk groups, the researchers reported positive agreement between IGRAs and the TST of 42.0%, negative agreement of 16.1%, and discordance of 41.9% [12]. Exploring the potential risk factors for discordance between IGRAs and the TST through logistic regression, researchers found that 85.1% of cases where the TST was positive and IGRA was negative were explained by BCG vaccination or migration; where the TST was negative and IGRA was positive, age explained 49.1% of the discordance [13]. Moreover, other researchers have concluded, through a systematic review of indeterminate IGRA cases, that 1 in 26 IGRA tests are indeterminate [14], which suggests that the test may still be reliable nonetheless.

In another systematic review and meta-analysis paper, the researchers reported that some studies in children found no significant difference between IGRAs and the TST in identifying LTBI, whereas other studies reported that IGRAs outperformed the TST. The review further suggested that some studies showed that the IGRA was not significantly different from the TST when identifying LTBI among immunocompromised patients [15]. While inconclusive, these studies suggest that IGRAs may be a useful method in identifying LTBI, although further studies are needed to elucidate their performance among various groups.

Regarding the diagnosis of LTBI, 2011 saw a significant development when the World Health Organization (WHO) endorsed the use of specific IGRAs, including the Qiagen QuantiFERON-Gold (QFT-G), QuantiFERON-TB Gold In-Tube (QFT-GIT), and Oxford Immunotec T-SPOT.TB (T-Spot) [3]. Since then, there have been upgrades to existing kits, such as the QuantiFERON-TB Gold Plus (QFT-Plus) from Qiagen, and new IGRAs have been added. Some of the additions include QIAreach (Qiagen), the Wantai TB-IGRA (TB-IGRA, Beijing Wantai Biological Pharmacy Enterprise), the STANDARD™ F TB-Feron FIA (TBF-FIA, SD Biosensor), and the STANDARD E TB-Feron ELISA (TBF-ELISA, SD Biosensor).

In this paper, we report the results of the evaluation of the diagnostic performance of the TBF-FIA, TBF-ELISA, and QFT-Plus among populations that are exposed or unexposed to MTB infection. This will contribute to the growing literature by providing evidence of the performance of these assays in an endemic country like Ghana.

## 2. Materials and Methods

### 2.1. Study Design

In this prospective, cross-sectional study, we evaluated the diagnostic performance of three IGRA kits: TBF-FIA, TBF-ELISA, and QFT-Plus. We also assessed how well the first two assays agreed with the established QFT-Plus test. We recruited a cohort of individuals suspected of having latent or active tuberculosis (TB), as well as individuals without known TB exposure, at the study’s onset. Each participant was tested using all three IGRA kits, enabling a direct and simultaneous comparison of the results across the assays. This approach ensured that the same patient population was used to assess each kit, providing a clear comparison of their diagnostic accuracy. By comparing the assays under the same conditions, this study design effectively minimized potential confounding factors and biases, allowing for a reliable evaluation of the relative performance of these diagnostic tools.

### 2.2. Study Site/Population

The National Public Health and Reference Laboratory (NPHRL), Ghana, conducted the testing due to its expertise and accreditation to ISO 15189 in various diagnostic fields. Samples were collected from eight TB clinics in proximity to the NPHRL in the Greater Accra, Eastern, and Central Regions to ensure same-day delivery and optimal sample quality. According to the National TB Control Programme, the TB incidence rate in 2022 was estimated at 133 per 100,000 population, representing a decline of about 17% from 2015.

### 2.3. Sample Size Determination

Sample sizes were determined on a convenience basis with guidance from the framework for the evaluation of new tests for tuberculosis infection by the WHO and the Foundation for Innovative New Diagnostics (FIND) [16]. In accordance with this framework, the study participants were representative of the general population as far as possible. This included the inclusion of very young and elderly individuals, those with more severe disease, and participants with comorbidities.

### 2.4. Study Initiation

Before starting the study, all personnel involved in the enrolment of patients were trained on the study protocol, including the criteria for inclusion, data collection tools, specimen collection, handling, and transportation. The laboratory personnel were also trained in specimen receipt and the assay procedures using training materials from the manufacturer. They demonstrated competence by performing a trial run as described in the instructions for use, including the use of the result analysis software from the manufacturer.

### 2.5. Participant Recruitment

This study enrolled healthy individuals with no known TB exposure or a very low likelihood of TB infection (unexposed group) and patients with clinically active TB (exposed group), as defined in the framework for the evaluation of new tests for tuberculosis infection [16]. Only patients meeting the case definition and providing informed consent were enrolled. Patients with conditions compromising their safety or data integrity were excluded. The required data included the demographics, specimen type, clinical diagnosis, test results, and collection date. Sample collection and testing commenced on 15 June 2023 and concluded on 31 March 2024.

Participants were recruited at the designated TB clinics by appointed coordinators, following the approved protocol. Demographic and clinical details were documented as per the protocol for each participant. Blood collection followed standard venipuncture procedures and was performed by trained personnel, with approximately 8 mL drawn into heparinized tubes. Specimens were stored at room temperature and transported within 16 h to the NPHRL by courier in puncture-resistant boxes, arriving on the same day as collection. Upon arrival, NPHRL staff checked the containers and paperwork for completeness and documented any discrepancies.

### 2.6. Measures to Minimize Bias

To minimize bias in the study, several measures were implemented. Samples were randomly collected from patients or subjects meeting the inclusion criteria to prevent selection bias, ensuring a representative and unbiased participant pool. Both positive and negative samples were tested in a random order to avoid systematic biases. The study also used a dual-group approach: one group performed the tests, while another managed the specimens, keeping the testing personnel blinded to the specimens’ status. This blinding was crucial in preventing any preconceived notions from affecting the results. These rigorous methods ensured the reliability and validity of the study’s findings.

### 2.7. Laboratory Testing

The TBF-ELISA, TBF-FIA, and QFT-Plus tests were performed in parallel, following the manufacturers’ instructions, under controlled conditions, using calibrated equipment certified by the Ghana Standards Authority.

The STANDARD™ E TB-FERON ELISA is a blood test that detects TB infection by measuring the immune response. Whole blood is drawn and divided into three tubes: a nil tube (baseline), a TB antigen tube containing proteins specific to *M. tuberculosis* (ESAT-6, CFP-10, and TB7.7), and a mitogen tube (positive control). If a person has been infected with TB, their T-cells will release interferon-gamma (IFN-gamma) upon encountering the TB antigens, which is then measured by the ELISA assay. The nil tube and mitogen tube help to ensure accurate test results. The optical density measurement from the ELISA runs were evaluated using the ELISA Report software (v1.5.6.66) supplied by the manufacturer. A report containing both qualitative and quantitative results is generated from the software. There are three qualitative result outcomes, namely ‘positive’, ‘negative’, or ‘indeterminate’. The quantitative results represent the estimate of the IFN-gamma concentration in IU/mL from a standard curve. The overall procedure from sample collection to results requires expertise and laboratory equipment for the performance of the ELISA assays and a time commitment of between 16 and 30 h.

The STANDARD™ F TB-FERON FIA utilizes the same blood collection tubes, antigens (ESAT-6, CFP-10, and TB7.7), and specimen preparation as the STANDARD™ E TB-FERON ELISA. However, it employs immunofluorescence technology with the STANDARD F analyzer to provide both the qualitative and quantitative measurement of interferon-gamma (IFN-γ) in the sample. In this study, the F2400 analyzer (software version: V001.045) was utilized. Aside from specimen preparation, the entire assay procedure was conducted using the analyzer, simplifying the workflow considerably. The estimated turnaround time from sample collection to obtaining results ranged between 16 and 26 h. The fourth-generation QuantiFERON-TB Gold Plus (QFT-Plus) test measures cell-mediated immunity to TB by quantifying the interferon-gamma (IFN-γ) released from the whole blood upon exposure to specific antigens. It utilizes two main tubes: TB1 and TB2. Both contain peptide antigens simulating proteins ESAT-6 and CFP-10, targeting CD4+ T-helper lymphocytes. However, TB2 includes additional peptides designed to stimulate CD8+ cytotoxic T lymphocytes. Similar to the TBF-ELISA, the optical density data were analyzed using the QFT TB Gold Plus software (v2.71.2, Build06). The conditions and requirements for the assay procedure were similar to those for the TBF-ELISA.

### 2.8. Data Management and Analysis

The study data were recorded in an Excel database. All data processing tasks and statistical analyses were performed using Jupyter Notebook 6.5.2 with Python 3 (ipykernel) and RStudio version 1.1.414 (with R version 3.4.3 (30 November 2017)). Standard diagnostic performance metrics (sensitivity, specificity, positive/negative predictive values, accuracy) were estimated with the associated 95% confidence intervals using exact binomial methods. Cohen’s kappa was estimated to determine the agreement and evaluated against standard criteria [17].

Additional performance metrics, such as Youden’s index [18], the diagnostic odds ratio, and the positive and negative likelihood ratios, were estimated with their usual meanings and interpretations implied [19].

## 3. Results

### 3.1. Participant Demographics

A total of 668 participants were recruited for the study; about 55.4% (370) were healthy individuals with no known TB exposure or a very low likelihood of TB infection (unexposed group) and 44.6% (298) were patients with clinically active TB (exposed group). When analyzing the sex of the participants, 61.5% were female and 38.3% were male; for one participant, the sex was missing. Additionally, the majority of the participants (42.8%) were recruited from Weija and 2.7% were recruited from Nsawam (Table 1).

The analysis of the age distribution of the participants, shown in Table 2, was based on 664 participants whose age was determined, and four participants were omitted due to a lack of information on their age. The analysis showed that the mean age of the female participants was 33.7 years (range: 0.8–75.0 years) and that of the male participants was 40.5 years (range: 2.0–80.0 years). On average, the exposed group was older (39.9 years) than the unexposed group (33.4 years). The youngest participant was 0.8 years old (9 months) and female, in the unexposed group, and recruited from the site in Weija. On the other hand, the oldest was an 80-year-old male, in the exposed group, and recruited from the site in Ashaiman. The analysis further demonstrated that the age distribution of the participants may generally reflect that of the target population.

### 3.2. Results of Assay Performance

The observed distribution of the results from each kit based on the case groups of participants are presented in the stacked contingency table, Table 3, below.

From the exposed group, the TBF-ELISA identified 92.3% as positive and 7.7% as negative. The TBF-FIA results identified 83.2% as positive and 16.8% as negative; for QFT-Plus, 88.9% of the exposed group were identified as positive and 11.1% were negative.

When analyzing the unexposed group, the TBF-ELISA identified 16.5% as positive and 83.5% as negative. The TBF-FIA, on the other hand, identified 13.2% of the unexposed group as positive and 86.8% as negative; the QFT-Plus results identified 11.9% of the unexposed participants as positive and 88.1% as negative.

The estimated performance characteristics for each assay with the associated 95% confidence intervals are presented in Table 4 below.

For the study population, the TBF-ELISA had the highest sensitivity estimate of 92.3% [95%CI: 88.6–95.0%] compared with the other two assays. Therefore, the probability of obtaining a positive test result among participants with TB infection is higher with the TBF-ELISA, followed by QFT-Plus (88.9% [95%CI: 84.8–92.3%]) and TBF-FIA (83.2% [95%CI: 78.5–87.3%]). Despite the differences, the overlap in the estimated confidence intervals for the three assays may suggest that the differences are not statistically significant. Alternatively, the probability of having a TB infection in a participant with a positive result, given as the positive predictive value, was higher for the QFT-Plus at 85.8% [95%CI: 81.4–89.5%] and lower in the TBF-ELISA at 81.8% [95%CI: 77.3–85.8%].

The probability of a negative test result in a participant without the disease was 88.1% [95%CI: 84.4–91.2%] when tested with the QFT-Plus, 86.8% [95%CI: 82.9–90.0%] when tested with the TBF-FIA, and 83.5% [95%CI: 79.3–87.1%] when tested with the TBF-ELISA. Again, the overlap in the confidence intervals may suggest that the differences in probability may not be statistically significant. However, the probability of not having a TB infection in a participant with a negative test result, given as the negative predictive value, is highest when tested with the TBF-ELISA (93.1% [95%CI: 89.8–95.6%]) and lowest when tested with the TBF-FIA (86.5% [95%CI: 82.6–89.8%]).

Consequently, the proportion of participants correctly classified by the assays (diagnostic accuracy) was estimated to be 88.5% [95%CI: 85.8–90.8%] for the QFT-Plus, which was the highest among the three assays, and the lowest was 85.2% [95%CI: 82.3–87.8%] for the TBF-FIA. The odds of a positive result in the exposed group relative to a positive result in the unexposed group was estimated as 60.6 [95%CI: 36.5–100.5] for the TBF-ELISA, 59.5 [95%CI: 36.8–96.1] for QFT-Plus, and 32.5 [95%CI: 21.2–49.8] for TFB-FIA. Despite the relatively higher diagnostic odds for the TBF-ELISA and QFT-Plus, their confidence intervals are quite wide, which may suggest less precision compared to the TBF-FIA, which has relatively lower odds but a narrow confidence interval.

Moreover, the probability of making an informed decision (rather than a random guess) when the assays are used in TB diagnosis, as given by the Youden index, was estimated as 0.77 [95%CI: 0.69–0.83] for QFT-Plus, 0.76 [95%CI: 0.68–0.82] for TBF-ELISA, and 0.70 [95%CI: 0.61–0.77] for TBF-FIA, suggesting that the utility of all three assays in decision-making regarding TB infection is comparable. A related measure, the number needed to diagnose, which represents the number of TB patients who need to be tested with the assays to correctly detect one person with a TB infection in the exposed and unexposed groups, was estimated as <2 for all three assays.

Additionally, the probability of obtaining a positive test result in the exposed group is 5.6 times higher than in the unexposed group when using the TBF-ELISA. When using the TBF-FIA, this probability is estimated at 6.3; for the QFT-Plus, the probability is 7.5. Further, the probability of obtaining a negative result in the exposed group is 0.09 times compared with the unexposed group for the TBF-ELISA, 0.19 times for the TBF-FIA, and 0.13 times for the QFT-Plus. These results indicate that the QFT-Plus may be relatively better when ruling-in a diagnosis, although its contribution to diagnosis may not be significant given that the estimated LR+ < 10. In contrast, the TBF-ELISA may be relatively better in ruling-out a diagnosis, and, given that the estimated LR− < 0.1, the contribution to lowering the posterior probability that the patient has the disease may be significant.

### 3.3. Agreement Analysis

The comparison of the results obtained when testing all three assays is summarized in Table 5. The analysis shows that, for 45.7% (305) of the tests run, all three assays were negative for the same samples. Conversely, all three assays were positive for the same samples in 39.2% (262) of the samples tested. Therefore, the proportion of samples for which all three assays reported the same results (negative/positive) was 84.9% (567).

Both the TBF-ELISA and QFT-Plus were only positive in 4.8% (32) of the samples tested and the TBF-ELISA was positive in only 3.6% (24) of the samples tested.

Consequently, using the QFT-Plus as the reference assay, the agreement with the TBF-ELISA and TBF-FIA was estimated. The contingency table of the observed values is presented in Table 6. The TBF-ELISA and QFT-Plus were both positive in 95.1% of the samples tested and negative in 88.3% of the samples. On the other hand, the TBF-FIA and QFT-Plus were positive in 86.4% of the samples tested and negative in 91.6% of the samples tested.

The overall agreement between the TBF-ELISA and QFT-Plus was estimated at 91.5%, with positive agreement of 95.2% and negative agreement of 88.3%. The strength of the agreement was estimated with Cohen’s kappa as 0.829, which can be evaluated as very high equivalence (Table 7). The overall agreement between the TBF-FIA and QFT-Plus was estimated as 89.2%, with positive agreement of 86.4% and negative agreement of 91.6%. The two assays have a high equivalence based on the Cohen’s kappa of 0.783.

## 4. Discussion

Although the WHO has made recommendations regarding the use of IGRAs in the diagnosis of TB infection [3], some studies have attempted to elucidate their utility in various clinical settings. For example, some researchers have suggested that, due to various factors, including limited data, the potential benefits of its use may be limited [20]. Other researchers have also suggested low accuracy as a limitation to the use of IGRAs [21].

In this study, we have reported that the sensitivity of the three assays lies within a combined 95% confidence interval of 78.5% to 95.0% and the specificity lies within 79.3% to 91.2%. These findings are comparable to what has been reported in the literature. Confidence intervals of 62.1% to 85.9% have been reported for the sensitivity and between 76.1% to 86.1% for the specificity of IGRAs [22].

We also report that the sensitivity of the TBF-ELISA in this study is about 3.4% higher than that of the QFT-Plus and the specificity is about 4.6% lower than that of the QFT-Plus, which are comparable to the findings in other studies, reporting that the sensitivity of the TBF-ELISA is 3.7% higher than that of the QFT and the specificity is 5.4% lower than that of the QFT-Plus [23]. Additionally, we report the kappa statistic for the agreement between the two assays as 0.83, which is comparable to the value of 0.85 reported elsewhere [23] or 0.9176 reported in another study [24].

We report the 95% confidence interval for the TBF-FIA’s sensitivity to be between 78.5% and 87.3%; the 95% confidence interval for the specificity was estimated to be 82.9% to 90.0%. Although there are limited data in the literature for the TBF-FIA, the findings can be viewed in relation to QIAreach, a similar device from Qiagen, whose sensitivity and specificity have been reported to be between 87.9% and 99.6% and 88.4% and 97.6%, respectively [24]. Another study reported the sensitivity and specificity of QIAreach as 98.5% and 72.3%, respectively [25]. The absence of overlap in the confidence intervals for the sensitivity may suggest that QIAreach has significantly higher sensitivity than the TBF-FIA; however, the specificity may not be significantly different. Moreover, the agreement between the TBF-FIA and QFT-Plus is high, with a kappa statistic of 0.783.

The higher sensitivity of the TBF-ELISA compared to the QFT-Plus observed in our study may suggest that the TBF-ELISA is a more sensitive tool for the detection of TB infection. However, its lower specificity may indicate a higher likelihood of false-positive results, which could lead to unnecessary treatment and increased healthcare costs. Despite these differences, both assays generally provide consistent results.

On the other hand, the differences in sensitivity between the TBF-FIA and QIAreach could be due to differences in the underlying technology or variations in the study populations. Therefore, further comparative studies are required to elucidate these differences.

Our findings suggest that the three IGRAs evaluated in this study (TBF-ELISA, TBF-FIA, and QFT-Plus) offer comparable diagnostic accuracy for TB infection. Additionally, the variability in the reported sensitivity and specificity values across different studies highlights the influence of factors such as the prevalence of TB, the population studied, and the cut-off values used for the interpretation of the assays. These factors need to be considered when interpreting the results of IGRAs in different clinical settings. Moreover, as indicated by some researchers, IGRAs cannot distinguish between latent and active TB, which limits their utility in diagnosing TB in highly endemic regions. This is particularly problematic in HIV-positive patients, where the accuracy of these tests is even more questionable due to the high rate of false negatives [20].

Despite the promising results observed in our study, several limitations must be acknowledged. Firstly, the diversity in the study population was not assessed, which may affect the generalizability of our findings. Future studies with more diverse populations are needed to confirm these results. Secondly, the analysis presented does not include indeterminate results. Such data may be invaluable in understanding the reliability and overall cost-effectiveness of the assays, as well as generating insights that can inform the development of next-generation IGRAs with improved performance and fewer indeterminate outcomes. Thirdly, the findings should not be interpreted to mean that a positive result with these kits indicates a latent TB infection. Such distinction may require the consideration of other variables, such as epidemiological linkages, longitudinal analysis, and the results of other tests. Finally, the further analysis of the immune response data from these assays could yield valuable insights. Although such analysis is beyond the scope of the current work, the researchers intend to present these findings in a separate publication.

## 5. Conclusions

This study adds to the growing body of evidence on the diagnostic accuracy of IGRAs. While our study provides valuable insights into the performance of different IGRAs, further research is necessary to fully understand their utility and limitations in various clinical contexts. Consequently, the continued evaluation and comparison of these assays will help to optimize their use in TB diagnosis and improve patient outcomes. Longitudinal studies to determine their utility in predicting the progression of the disease are urgently needed.

## Figures and Tables

**Table 1:** Number of participants recruited by case group, sex, and location.

Variable	Frequency	Percent
**Group**		
Unexposed	370	55.4%
Exposed	298	44.6%
**Sex**		
Female	411	61.5%
Male	256	38.3%
NA	1	0.2%
**Location**		
Weija	286	42.8%
Ridge	87	13.0%
Kasoa	59	8.8%
Ga West	55	8.2%
Tema	54	8.1%
Ashaiman	49	7.3%
Achimota	32	4.8%
Korle Bu	28	4.2%
Nsawam	18	2.7%
Total	668	100%

**Table 2:** Statistical distribution of participant age (in years) by sex, case group, and location.

Variable	Mean	SD	Median	Min	Max	Range	Skew	Kurtosis	SE
**Sex**									
Female	33.7	12.1	32.0	0.8	75.0	74.3	0.9	1.0	0.6
Male	40.5	14.4	41.0	2.0	80.0	78.0	0.0	−0.1	0.9
**Group**									
Unexposed	33.4	13.2	31.0	0.8	75.0	74.3	0.8	1.0	0.7
Exposed	39.9	12.9	39.0	4.0	80.0	76.0	0.3	−0.3	0.8
**Location**									
Achimota	37.4	10.4	35.5	23.0	54.0	31.0	0.2	−1.4	1.8
Ashaiman	36.4	13.4	33.0	14.0	80.0	66.0	0.8	0.6	1.9
Ga West	41.3	12.9	40.0	11.0	71.0	60.0	0.2	−0.4	1.7
Kasoa	31.7	11.1	29.5	17.0	67.0	50.0	1.1	0.8	1.5
Korle Bu	38.2	13.9	39.0	21.0	65.0	44.0	0.4	−1.1	2.6
Nsawam	35.8	18.3	34.0	13.0	75.0	62.0	0.8	−0.4	4.3
Ridge	40.1	13.8	40.0	4.0	75.0	71.0	0.1	−0.2	1.5
Tema	40.3	11.8	42.0	16.0	65.0	49.0	0.0	−1.0	1.6
Weija	34.0	13.4	32.0	0.8	74.0	73.3	0.7	0.8	0.8

**Table 3:** Observed distribution of assay results by case group.

Kit Name	Exposed (%)	Unexposed (%)	Total
**TBF-ELISA**			
Positive	275 (92.3%)	61 (16.5%)	336
Negative	23 (7.7%)	309 (83.5%)	332
**TBF-FIA**			
Positive	248 (83.2%)	49 (13.2%)	297
Negative	50 (16.8%)	321 (86.8%)	371
**QFT-Plus**			
Positive	265 (88.9%)	44 (11.9%)	309
Negative	33 (11.1%)	326 (88.1%)	359
Total	298	370	668

**Table 4:** Estimates of performance characteristics per assay.

	TBF-ELISA	TBF-FIA	QFT-Plus
Statistic	Estimate [95% CI]	Estimate [95% CI]	Estimate [95% CI]
Sensitivity	92.3% [88.6–95.0%]	83.2% [78.5–87.3%]	88.9% [84.8–92.3%]
Specificity	83.5% [79.3–87.1%]	86.8% [82.9–90.0%]	88.1% [84.4–91.2%]
Positive predictive value	81.8% [77.3–85.8%]	83.5% [78.8–87.5%]	85.8% [81.4–89.5%]
Negative predictive value	93.1% [89.8–95.6%]	86.5% [82.6–89.8%]	90.8% [87.3–93.6%]
Diagnostic accuracy	87.4% [84.7–89.8%]	85.2% [82.3–87.8%]	88.5% [85.8–90.8%]
Diagnostic odds ratio	60.6 [36.5–100.5]	32.5 [21.2–49.8]	59.5 [36.8–96.1]
Youden index	0.76 [0.68–0.82]	0.70 [0.61–0.77]	0.77 [0.69–0.83]
Positive likelihood ratio	5.6 [4.4–7.1]	6.3 [4.8–8.2]	7.5 [5.7–9.9]
Negative likelihood ratio	0.09 [0.06–0.14]	0.19 [0.15–0.25]	0.13 [0.09–0.17]
Number needed to diagnose	1.3 [1.2–1.5]	1.4 [1.3–1.6]	1.3 [1.2–1.4]

**Table 5:** Joint distribution of results of three assays.

Definition	Frequency	Percent
None of the tests are positive	305	45.7%
Only QFT-Plus is positive	10	1.5%
Only TBF-FIA is positive	12	1.8%
Both TBF-FIA and QFT-Plus are positive	5	0.7%
Only TBF-ELISA is positive	24	3.6%
Both TBF-ELISA and QFT-Plus are positive	32	4.8%
Both TBF-ELISA and TBF-FIA are positive	18	2.7%
All three tests are positive	262	39.2%
Total	668	100

**Table 6:** Crosstabulation of QFT-Plus with TB-ELISA and TBF-FIA.

		QFT-Plus		
		Positive (%)	Negative (%)	Total
**TBF-ELISA**	Positive	294 (95.1%)	42 (11.7%)	336
	Negative	15 (4.9%)	317 (88.3%)	332
**TBF-FIA**	Positive	267 (86.4%)	30 (8.4%)	297
	Negative	42 (13.6%)	329 (91.6%)	371
	**Total**	**309**	**359**	**668**

**Table 7:** Estimates of agreement using QFT-Plus as reference assay.

	TBF-ELISA/QFT-Plus	TBF-FIA/QFT-Plus
Statistic	Estimate	Estimate
Overall agreement	91.5%	89.2%
Positive agreement	95.2%	86.4%
Negative agreement	88.3%	91.6%
Kappa	0.829	0.783

## Data Availability

The datasets presented in this article are not readily available because the data are part of an ongoing study. Requests to access the datasets should be directed to the corresponding author.
